# Developmental assessment of children with intrauterine exposure to Zika virus: cross-sectional observational study

**DOI:** 10.17843/rpmesp.2023.403.12880

**Published:** 2023-09-26

**Authors:** Victor H. Estupiñan-Perez, Angela M. Jiménez-Urrego, Freiser E. Cruz-Mosquera, Alejandro Botero-Carvajal

**Affiliations:** 1 Health Faculty, Universidad Santiago de Cali, Cali, Colombia. Universidad Santiago de Cali Health Faculty Universidad Santiago de Cali Cali Colombia; 2 Universidad de San Buenaventura, Cali, Colombia. Universidad de San Buenaventura Universidad de San Buenaventura Cali Colombia

**Keywords:** Zika Virus Infection, Developmental Assessment, Observational Study, Child Development, Neuropsychological Tests

## Abstract

Zika virus infection affects the development of the nervous system. This study describes the cognitive, adaptative, communicative, social and motor neurodevelopment of children exposed to Zika virus in utero. We used the Batelle scale to assess neurodevelopment three years after birth. Thirty children were included, who had a mean age at evaluation of 37.5 (IQR: 35.7-39.2) months. We found the following equivalent ages in months for each area: motor 25.8 (SD: 7.8), adaptive 26.7 (SD: 5.8), communicative 30.2 (SD: 6.9), social personal 33.5 (SD: 8.3) and cognitive 35.6 (SD: 5.9). Children showed development delay for their chronological age, 25 children were delayed in one of the five areas assessed. A high rate of children exposed to Zika virus during gestation presented delayed developmental age, mainly regarding the adaptive and motor areas.

## INTRODUCTION

Zika virus arrived to the Americas in 2015 and was reported in about 57 countries during the next two years ^1^^,^^2^. In pregnant women, the virus affects the health of the mother and fetus ^3^^,^^4^. The virus is transmitted vertically in 20% to 30% of the cases, which is referred to as “Zika virus-associated congenital syndrome” and causes defects in the fetus and newborn, although half are asymptomatic ^3^. Between 4% to 6% of infected fetuses develop microcephaly, with visible adverse consequences in early childhood; about 47% of children exposed to the virus have neurodevelopmental abnormalities ^5^. Exposure is associated with cognitive language delays in development, even in normocephalic children ^6^.

Leboy *et al*. ^7^ reported that the virus replicates in tissues, such as the brain. The alterations caused by neurotropism affect neuronal differentiation, causing functional and morphological alterations; in addition, it causes apoptosis of neural progenitor cells, which is one of the causes of microcephaly and neurodevelopmental alterations, causing subtle delays in language, cognition, motor and visual domains that together with malformations of cortical development contribute to cognitive and intellectual deficits and learning disorders ^7^^-^^9^.

Hicini *et al*. ^10^, in a cohort of 129 children, found that infants with congenital Zika-associated syndrome presented neurological impairment and motor component delay more frequently; in addition, they had a fourfold greater probability of neurodevelopmental delay at three years of age. Another follow-up at 11 and 32 months showed that children exposed intrauterine to Zika virus have a higher risk of mild cognitive delay and a higher probability of impaired auditory behavior ^11^. Similarly, Stringer *et al*. ^12^ evaluated 129 children with no evidence of Zika-associated congenital syndrome up to 24 months, and they found low scores on neurocognitive assessment in those exposed.

In Colombia, there are few studies on the follow-up of children’s development after exposure to Zika virus. Research shows the need for attention and actions to enhance development, defined as the expression of skills in the following dimensions: personal/social, adaptive, motor, communicative and cognitive ^(^^7^. This study aimed to describe the neurodevelopment of children exposed intrauterine to Zika virus three years after birth, in the city of Palmira in Colombia.

**KEY MESSAGES**

Motivation for the study. Little is known about the effect of Zika virus exposure on neurodevelopment of intrauterine exposed infants.

Main findings. The virus affects neurodevelopment in all five dimensions assessed. The highest percentage of children at risk was found in two of the five areas: motor and communication. This impairment delays psychosocial and cognitive development.

Implications. The results reinforce that Zika virus infection can produce an important economic burden for the health system, due to the sequelae found in children exposed during the gestation period. It is important that surveillance systems allow the identification and strict follow-up of this population in order to detect problems in their development.

## THE STUDY

### Design and participants

A cross-sectional study was conducted between July and October 2019, on children residing in the rural area of the city of Palmira, Valle del Cauca, in Colombia, where a Zika outbreak occurred in previous years. Mothers with positive serological tests during pregnancy were included. These pregnant women were registered in a database provided by the Departmental Health Secretariat of Valle del Cauca after the epidemic. Children exposed to the virus intrauterinally were evaluated three years after birth. We obtained data regarding the place of residence and the telephone numbers that allowed the respective contact from the records. Of the 122 children, 30 were included in the final evaluation; the rest were not included due to difficulties related to telephone contact, changes in the place of residence and problems regarding access to their home. Children whose parents decided not to participate in the research were excluded.

### Variables and instrument

The following sociodemographic variables were included: age, sex, affiliation to the General Social Security Health System (SGSSS) and socioeconomic level, according to the National Administrative Department of Statistics of Colombia, which classifies the economic condition based on the location of the property (1 is considered the lowest stratum and 6 the highest). On the other hand, anthropometric variables were included, such as current weight (kg), birth weight (kg), current height (cm) and height at birth (cm). Information regarding maternal medical history was also included, such as history of herpes, HELLP syndrome, preeclampsia, eclampsia and Zika-associated symptoms such as arthralgia, skin rash, myalgia and conjunctivitis.

We used the Battelle scale ^13^ to assess children’s development. The instrument has 341 items grouped into five areas: personal/social (capacities and characteristics that allow the child to establish meaningful social interactions), adaptive (capacities and characteristics of the child to be independent and assume personal responsibility for his/her actions), motor (capacities for gross and fine motor control), communication (assesses the reception and expression of information by verbal and nonverbal means) and cognitive (conceptual skills and capacities). Each area scored between 0 and 100. Values between 0 and 34 were considered as “risk”, between 35 and 65 were considered as “normal” and between 66 and 100 were considered as “strength”.

### Data collection

Parents were contacted by telephone to share the information and inquire about their willingness to participate. After obtaining consent, a psychologist expert in child development went to the home of each participant, applied the scale and socialized the results of the test.

### Statistical analysis

Data were analyzed in SPSS version 24 software. We identified missing and extreme values with an exploratory analysis. The Shapiro Wilk test was used to determine the normality of the distribution of quantitative variables. Categorical variables were reported with frequencies and percentages, quantitative variables with median and interquartile range (IQR) or mean and standard deviation (SD), depending on their distribution.

### Ethical considerations

This research followed the guidelines of the Ministry of Health and Social Protection of Colombia and the Declaration of Helsinki. The protocol had the ethical endorsement of the Universidad Santiago de Cali, through the document of session No. 12 and the authorization of the Health Secretariat of Palmira. The parents of the participants filled out the parental consent form and all data derived from the research were treated confidentially.

## FINDINGS

Of the 30 participants, 53.3% were male, the median age was 37.5 months (IQR: 35.7-39.2), most were affiliated to the SGSSS through the contributory regime (93.3%), 13.3% of the children were premature, and 6.7% presented microcephaly. Regarding anthropometric characteristics, the median height and birth weight were 50 cm (IQR: 47-52) and 3 kg (IQR: 2.7-3.4), respectively; with a body mass index of 12.3 kg/m^2^ (IQR: 11.6-13.5) (Table 1).

**Table 1:** Sociodemographic and anthropometric characteristics of the population studied (n=30).

Variable	n	%
Sex		
Female	14	46.7
Male	16	53.3
SGSSS affiliation		
Contributive	28	93.3
Subsidized	2	6.7
Socioeconomic level		
1	2	6.7
2	16	53.3
3	4	13.3
4	8	26.7
5	0	0.0
6	0	0.0
Gestational week at birth		
Preterm	4	13.3
Full term	26	86.7
Post-term	0	0.0
Microcephaly at birth		
Yes	2	6.7
No	28	93.3
Current age (months) ^a^	37.5	35.7-39.2
Birth weight (kg) ^a^	3	2.7-3.4
Size at birth (cm) ^a^	50	47-52
BMI at birth (Kg/m^2^) ^a^	12.3	11.6-13.5
Current weight (kg) ^a^	15	13-17
Current size (cm) ^a^	95	91-99
Current BMI (Kg/m^2^) ^a^	15.3	13.8-19.6

a Median and interquartile range.

SGSSS: General Social Security Health System, BMI: body mass index.

On the other hand, women did not have history of eclampsia, syphilis or HELLP syndrome, 6.7% reported having presented preeclampsia and 3.3% herpes. The most frequently reported clinical manifestations were skin rash (96.7%), arthralgia (56.7%) and myalgia (53.3%) (Table 2).

**Table 2:** Symptomatology and medical history of mothers diagnosed with Zika (n=30).

Variable	n	%
Symptomatology		
Arthralgia		
Yes	17	56.7
No	13	43.3
Rash		
Leve	10	33.3
Moderate	6	20.0
Severe	13	43.3
No	1	3.4
Conjunctivitis		
Yes	10	33.3
No	20	66.7
Myalgia		
Yes	16	53.3
No	14	46.7
Background		
Preeclampsia		
Yes	2	6.7
No	28	93.3
Eclampsia		
Yes	0	0.0
No	30	100
HELLP syndrome		
Yes	0	0.0
No	30	100
Syphilis		
Yes	0	0.0
No	30	100
Herpes		
Yes	1	3.3
No	29	96.7

**Figure 1:**
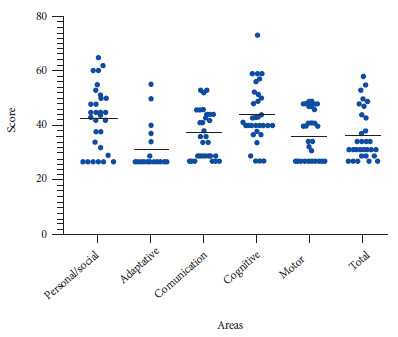
Scores by developmental area according to Battelle’s scale

The development areas with the highest mean scores were cognitive with 44 (SD: 10), personal/social with 42 (SD: 11) and communication with 37 (SD: 8.7) (Figure 1).

The above scores show that, in two of the five areas, children at risk were more frequent than children with normality and strength. The development areas with the highest percentage of children at risk were: adaptive (80%), motor (53%) and communication (43%). Only 3% of the participants presented strengths in the cognitive area (Supplementary Material).

The developmental assessment showed that 25 children presented delayed development in one of the five areas assessed. The mean age equivalent for the motor, adaptive, communicative, personal/social and cognitive areas, respectively, were: 25.8 (SD: 7.8) months, 26.7 (SD: 5.8) months, 30.2 (SD: 6.9) months, 33.5 (SD: 8.3) months and 35.6 (SD: 5.9) months. The adaptive domain was the most affected and the least affected one was the cognitive domain (Table 3).

**Table 3:** Description and average of age in months for each area according to the results of the Battelle scale (n=30).

Current age (months)	Cognition	Communication	Motor	Adaptative	Personal/social
	(Equivalent age in months)	(Equivalent age in months)	(Equivalent age in months)	(Equivalent age in months)	(Equivalent age in months)
45	46	39	42	26	46
41	23	7	8	10	12
41	41	36	40	33	41
41	42	39	37	33	42
40	38	39	30	25	44
40	43	31	25	24	39
40	35	27	24	30	38
39	22	22	14	22	22
39	38	36	27	33	39
39	39	23	31	26	33
38	42	36	25	30	33
38	36	33	25	28	35
38	33	27	25	30	24
38	35	35	26	26	36
38	37	34	34	22	35
37	36	35	24	32	37
37	35	38	17	25	31
37	36	29	29	26	37
37	33	28	17	23	30
37	31	22	24	25	26
37	36	32	27	26	36
36	34	33	29	34	27
36	35	32	31	27	30
35	44	24	29	34	39
35	37	27	26	28	39
34	35	32	17	24	40
34	20	21	8	11	12
33	31	25	26	34	28
32	37	32	28	31	33
32	37	32	29	24	43
37.4 (2.9) ^a^	35.6 (5.9) ^a^	30.2 (6.9) ^a^	25.8 (7.8) ^a^	26.7 (5.8) ^a^	33.5 (8.3) ^a^

a Mean and standard deviation.

## DISCUSSION

This study describes the neurodevelopmental status of 30 children at 36 months of exposure to Zika virus. The scores suggest that, on average, the children presented an equivalent age in months below their actual chronological age. According to the scores, 80% of the children presented the highest developmental risk regarding the adaptive domain.

Issues regarding motor and adaptive development mean that children, three years after birth, show difficulties in being independent, assuming responsibility for their actions and in gross and fine motor control. Both aspects are interdependent, since motor skills allow for greater independence and responsibility ^14^. We believe that this is related to the lack of skills and resources of mothers to stimulate their children, once the diagnosis is established. This is due to the fact that sensory-motor integration, as well as gross and fine motor stimulation, depends on the accompaniment and feedback that the caregiver can offer, together with the specialized intervention of physiotherapy or occupational therapy which, in our case, was late ^7^^,^^9^.

However, the risk reported in the motor area does not seem to affect the cognitive performance of the participants. We believe that, the little effect that the virus has on the cognitive dimension (formation of categories and concepts), is due to the stimulation activities accessible in the mother-child interaction, such as: perceptual discrimination, identifying and solving problems, assessing contradictions and inconsistencies ^15^ proposed by most games for children from 0 to 5 years of age ^16^.

The Battelle scale has been culturally adapted in Colombia, with high interobserver reliability ^13^. It has also been used in Colombia, where it showed adequate psychometric properties ^17^. Although it is one of several options for measuring neurodevelopment, and is of high clinical interest with multiple practical applications, it is susceptible to comparison with other instruments that evaluate the same construct, and each one has broad cut-off points that allow an adequate identification of neurodevelopment ^18^. Our results compared with other studies on other diseases suggest a severe developmental impact associated with Zika ^13^^,^^14^.

Although we recognize that case-control or cohort studies are needed to analyze the effect of the association between neurodevelopmental alterations and Zika, our descriptive results show alterations in exposed children. It is important to consider the time of measurement when analyzing neurodevelopment; our evaluation occurred at 36 months, with results different from studies that considered 24 months ^19^, which reported uneven patterns in the dimensions. For example, significant associations were found between maternal exposure and Zika in the personal/social and problem-solving areas, but the comparison between children exposed and not exposed to the virus showed similar levels of motor development, problem-solving and personal and social skills. Even language proficiency was mostly affected in children not exposed to the virus compared to those exposed: 20.3% versus 8.3% in those not exposed ^16^. This contradiction underlines the idea of the interaction between dimensions and the environmental factor on brain maturation ^20^.

One limitation of the study is related to the lack of information from diagnosis to subsequent neurodevelopmental progression, because environmental characteristics (socioeconomic level, income, parenting styles, affective or cultural stimulation or deprivation, formal education) interact with biological factors and individual characteristics to produce the outcomes assessed by the instrument. Another limitation was the high social mobility of the Colombian population, since, we could only contact 30 of the 122 children registered in the database. However, our strength is that this is one of the few studies that performs neurodevelopmental assessment in patients exposed to the Zika virus in Colombia. However, we recognize that including a control group could have helped with the evaluation of neurodevelopment in sociodemographic, nutritional, cultural and family conditions similar to the evaluated group, which would facilitate identifying the role of the virus on neurodevelopment. However, we found studies that explore the association between neurodevelopment and the virus without a control group and with a low number of cases ^21^^,^^22^.

Likewise, neurodevelopment is a multicausal and complex process in which biological and sociocultural factors interact. Some biological characteristics such as nutritional status, intestinal parasitosis and malaria may have affected neurodevelopment in our sample. In turn, sociocultural aspects such as educational or parental stimulation were not evaluated in this study. These characteristics may explain the developmental status of children in our sample; therefore, our results should be interpreted with these limitations in mind. However, comparable follow-up studies of children exposed to Zika virus included similar variables (anthropometric variables, current weight, birth weight, current height and height at birth), which are considered as a measure of the biological conditions of the child at the time of evaluation ^22^^,^^23^.

One factor that may modulate motor neurodevelopment is the increased use of digital devices. For example, the younger the age of digital device use, the greater the errors in motor performance tasks. However, this relationship is not very significant due to the effect of age on the relationship between digital device use and neuromotor development ^24^, therefore, it would be advisable to carry out a longitudinal follow-up of children taking into account the use of the mobile device, characteristics of stimulation, nutritional status and reported clinical conditions and age.

In our study, exposed children showed insufficient development in the third year of life. Since this study describes the development of exposed children in Colombia, follow-up studies are needed to corroborate these findings. Early diagnosis in pregnant women will allow early interventions by the health system, focused on vulnerable populations. This depends on the timeliness of diagnosis, due to the short period for virus detection being necessary to have early results. Follow-up during early childhood is crucial to detect possible neurological disorders, which can be diagnosed during primary care consultations, and early interventions by specialists can avoid repercussions that may affect neurodevelopment.

Although the number of Zika cases has decreased significantly, the infection continues to spread in some countries in the Americas ^21^. As a result, health services are likely to be overwhelmed in areas where there is still a high number of cases. It is important that caregivers have access to neurodevelopmental screening tools in areas where health systems have limited resources.

In conclusion, 30 children exposed intrauterinally to Zika virus showed neurodevelopmental delay, particularly in the motor and adaptive areas. Neurodevelopment is a process of integration and interaction between biological and environmental dimensions; therefore, interventions should be guided by caregivers and health personnel and aimed at strengthening motor and adaptive skills, which are dimensions of neurodevelopment that enable exploration and autonomy of the child.
