# Supplementary material for: Developmental assessment of children with intrauterine exposure to Zika virus: cross-sectional observational study
Source: Rev Peru Med Exp Salud Publica. 2023 Sep 26;40(3):333–9. doi: 10.17843/rpmesp.2023.403.12880 (PMC10959516; doi:10.17843/rpmesp.2023.403.12880)
Supplement: Supplementary material. — Available in the electronic version of the RPMESP. [file rpmesp-40-03-12880-s001.docx]

Material suplementario.


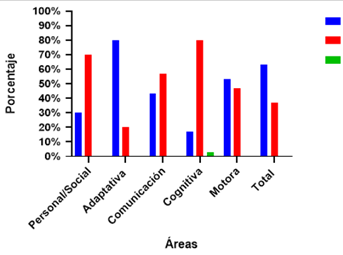


Azul: riesgo

Rojo: normalidad

Verde: fortaleza

**Anexo 1.** Porcentaje de niños en riesgo, normalidad y fortaleza según la escala de Battelle.
