# Supplementary material for: Developmental assessment of children with intrauterine exposure to Zika virus: cross-sectional observational study
Source: Rev Peru Med Exp Salud Publica. 2023 Sep 26;40(3):333–9. doi: 10.17843/rpmesp.2023.403.12880 (PMC10959516; doi:10.17843/rpmesp.2023.403.12880)
Supplement: Translation

# Translation: Evaluación del neurodesarrollo en niños con exposición intrauterina al virus del Zika: estudio observacional transversal

**Authors:** Victor H. Estupiñan-Perez, Angela M. Jiménez-Urrego, Freiser E. Cruz-Mosquera, Alejandro Botero-Carvajal

**Affiliations:** 1 Facultad de Salud, Universidad Santiago de Cali, Cali, Colombia.; 2 Universidad de San Buenaventura, Cali, Colombia.

**Keywords:** Infección por el virus del Zika, Evaluación del Desarrollo, Estudio Observacional, Desarrollo Infantil, Pruebas Neuropsicológicas

## Abstract

El virus del Zika produce desenlaces adversos para el desarrollo del sistema nervioso. Este estudio describe el neurodesarrollo cognitivo, adaptativo, comunicativo, social y motor de niños expuestos intrauterinamente al virus del Zika y hace una evaluación del neurodesarrollo con la escala de Battelle a los tres años después del nacimiento. Participaron 30 niños con una media de edad al momento de la evaluación de 37,5 (RIC: 35,7-39,2) meses. Se halló una edad equivalente en meses en las áreas: motora 25,8 (DE: 7,8), adaptativa 26,7 (DE: 5,8), comunicativa 30,2 (DE: 6,9), personal social 33,5 (DE: 8,3) y cognitiva 35,6 (DE: 5,9). Los niños presentaron retraso en el desarrollo para la edad cronológica, 25 niños presentaban retraso en una de las cinco áreas evaluadas. Una alta proporción de niños expuestos al virus del Zika durante la gestación presentaron retraso en el desarrollo, principalmente en el dominio adaptativo y motor.

## INTRODUCCIÓN

El virus del Zika llegó a las Américas en 2015 y se reportó en cerca de 57 países, dos años después ^1^^,^^2^. En mujeres embarazadas, el virus compromete la salud de la madre y del feto ^3^^,^^4^. El virus se transmite de forma vertical en un 20% a 30%, denominado «Síndrome congénito asociado al virus del Zika» con defectos en el feto y recién nacido, aunque la mitad son asintomáticos ^3^. Entre el 4% al 6% de los fetos infectados desarrollan microcefalia, con consecuencias adversas visibles en la primera infancia, cerca del 47% de los niños expuestos al virus tienen anomalías en el neurodesarrollo ^5^. La exposición se asocia a retrasos cognitivos del lenguaje, incluso en niños normocefálicos ^6^.

Leboy *et al.*^7^ refieren que el virus se replica en los tejidos incluido el cerebro. Las alteraciones causadas por el neurotropismo afectan la diferenciación neuronal, con alteraciones funcionales y morfológicas, además, provoca apoptosis de las células progenitoras neurales, una de las causas de microcefalia y alteraciones del neurodesarrollo, ocasionando retrasos sutiles en el lenguaje, cognición, dominios motores y visuales que junto a malformaciones del desarrollo cortical contribuyen a déficit cognitivo, intelectual y trastornos del aprendizaje ^7^^-^^9^.

Hicini *et al*. ^10^ en una cohorte de 129 niños encontraron que lactantes con el Síndrome congénito asociado al Zika, con mayor frecuencia presentan deterioro neurológico y retraso en el componente motor; además, una probabilidad cuatro veces mayor de retraso en el desarrollo neurológico a los tres años. Otro seguimiento a los 11 y 32 meses mostró que los niños expuestos intrauterinamente al virus del Zika tienen mayor riesgo de retraso cognitivo leve y una probabilidad más alta de conducta auditiva alterada ^11^. De igual forma, Stringer *et al*. ^12^ evaluó a 129 niños sin evidencia de síndrome congénito asociado al Zika hasta los 24 meses, encontró bajas puntuaciones en la valoración neurocognitiva en los expuestos.

En Colombia, existen pocos trabajos publicados de seguimiento a niños en su desarrollo luego de la exposición al virus del Zika. Los estudios publicados muestran la necesidad de atención y de acciones para potenciar el desarrollo, entendido como la expresión de habilidades en las dimensiones: personal/social, adaptativa, motora, comunicativa y cognitiva ^7^. El objetivo del estudio fue describir el neurodesarrollo de niños expuestos intrauterinamente al virus del Zika tres años del nacimiento, en la ciudad de Palmira en Colombia.

**MENSAJES CLAVE**

Motivación para realizar el estudio. Poco se sabe de la exposición del virus del Zika en el neurodesarrollo de los niños expuestos intrauterinamente.

Principales hallazgos. El virus afecta el neurodesarrollo en las cinco dimensiones evaluadas. El mayor porcentaje de niños en riesgo se encontró en dos de las cinco áreas evaluadas: motora y comunicación. Esta afectación retrasa el desarrollo psicosocial y cognitivo.

Implicaciones. Los resultados refuerzan que la infección por el virus del Zika puede producir una importante carga económica para el sistema de salud, debido a las secuelas encontradas en los niños expuestos durante el periodo de gestación. Es importante que los sistemas de vigilancia permitan identificar y realizar un estricto seguimiento a esta población con el fin de evidenciar problemas en su desarrollo.

## EL ESTUDIO

### Diseño y participantes

Se realizó un estudio de corte transversal entre julio y octubre del 2019, en el que participaron niños que residían en la zona rural de la ciudad de Palmira, Valle del Cauca, en Colombia, en donde se presentó un brote de zika en años anteriores. Se incluyeron madres con pruebas serológicas positivas durante el embarazo. Dichas embarazadas estaban registradas en una base de datos proporcionada por la Secretaría de Salud departamental del Valle del Cauca, posterior a la epidemia. Los niños expuestos al virus intrauterinamente fueron evaluados tres años después del nacimiento. A partir de los datos consignados se obtuvo el lugar de residencia y los números telefónicos que permitieron el contacto respectivo. De los 122 niños, se incluyeron 30 en la evaluación final, debido a: dificultades en el contacto telefónico, modificaciones en el lugar de residencia y problemas de acceso al domicilio. Se excluyeron niños cuyos padres decidieron no participar de la investigación.

### Variables e instrumento

Se contemplaron variables sociodemográficas: edad, sexo, régimen de afiliación al Sistema General de Seguridad Social en Salud (SGSSS) y nivel socioeconómico, según el Departamento Administrativo Nacional de Estadística de Colombia clasifica la condición económica a partir de la ubicación del inmueble (considera 1 como menor estrato y 6 como mayor). Por otro lado, se incluyeron las variables antropométricas: peso actual (kg), peso al nacer (kg), talla actual (cm) y talla al nacer (cm). Se tuvieron en cuenta antecedentes maternos: herpes, síndrome de HELLP, preeclampsia, eclampsia y sintomatología asociada a zika como artralgia, *rash* cutáneo, mialgia y conjuntivitis.

Para evaluar el desarrollo de los niños se empleó la escala de Battelle, utilizado en el tamizaje y evaluación del desarrollo infantil ^13^. El instrumento cuenta con 341 ítems agrupados en cinco áreas: personal/social (capacidades y características que permiten al niño establecer interacciones sociales significativas), adaptativa (capacidades y características del niño para ser independiente y asumir responsabilidades personales en sus acciones), motora (capacidades para el control motor grueso y fino), comunicación (valora la recepción y expresión de información por medios verbales y no verbales) y cognitiva (habilidades y capacidades de tipo conceptual). Cada área puntúa entre 0 a 100. Entre 0 y 34 es «riesgo», entre 35 y 65 es «normalidad» y entre 66 y 100 es «fortaleza».

### Recolección de los datos

Se contactó telefónicamente a los padres para socializar la información e indagar la voluntad de participación. Tras recibir el consentimiento, una psicóloga experta en desarrollo infantil se desplazó al domicilio de cada participante, aplicó el inventario y socializó los resultados de la prueba.

### Análisis estadístico

Los datos se analizaron en el software SPSS versión 24. Se realizó un análisis exploratorio para identificar valores omitidos y extremos. Para determinar la normalidad de la distribución de las variables cuantitativas se utilizó la prueba de Shapiro Wilk. Las variables categóricas se reportaron con frecuencias y porcentajes, las variables cuantitativas con la mediana y rango intercuartílico (RIC) o media y desviación estándar (DE), dependiendo de su distribución.

### Consideraciones éticas

La investigación siguió los lineamientos del Ministerio de salud y Protección Social de Colombia y de la Declaración del Helsinki. El protocolo contó con el aval ético de la Universidad Santiago de Cali, a través del acta de sesión N.º 12 y la autorización de la secretaría de Salud Palmira. Los padres de los participantes diligenciaron el consentimiento parental y los datos derivados de la investigación fueron tratados de forma confidencial.

## HALLAZGOS

De los 30 participantes, el 53,3% corresponde al sexo masculino, la mediana de edad fue de 37,5 meses (RIC: 35,7-39,2), en su mayoría afiliados al SGSSS a través del régimen contributivo (93,3%), el 13,3% de los niños fueron prematuros, el 6,7% presentó microcefalia. Respecto a las características antropométricas, se encontró una mediana de talla y peso al nacer de 50 cm (RIC: 47-52) y 3 kg (RIC: 2,7-3,4), respectivamente; con un índice de masa corporal de 12,3 Kg/m^2^ (RIC:11,6-13,5) (Tabla 1).

**Tabla 1:** Características sociodemográficas y antropométricas de la población estudiada (n=30).

Variable	n	%
Sexo		
Femenino	14	46,7
Masculino	16	53,3
Régimen de afiliación al SGSSS		
Contributivo	28	93,3
Subsidiado	2	6,7
Nivel socioeconómico		
1	2	6,7
2	16	53,3
3	4	13,3
4	8	26,7
5	0	0,0
6	0	0,0
Semana gestacional al nacer		
Pretérmino	4	13,3
A término	26	86,7
Postérmino	0	0,0
Microcefalia al nacer		
Sí	2	6,7
No	28	93,3
Edad actual (meses) ^a^	37,5	35,7-39,2
Peso al nacer (kg) ^a^	3	2,7-3,4
Talla al nacer (cm) ^a^	50	47-52
IMC al nacer (Kg/m^2^) ^a^	12,3	11,6-13,5
Peso actual (kg) ^a^	15	13-17
Talla actual (cm) ^a^	95	91-99
IMC actual (Kg/m^2^) ^a^	15,3	13,8-19,6

a Mediana y rango intercuartílico.

SGSSS: Sistema General de Seguridad Social en Salud, IMC: índice de masa corporal.

Por otra parte, no se encontró antecedentes de eclampsia, sífilis o síndrome de HELLP en las madres estudiadas, el 6,7% manifestó haber presentado preeclampsia y el 3,3% herpes. Las manifestaciones clínicas reportadas con mayor frecuencia fueron *rash* cutáneo (96,7%), artralgia (56,7%) y mialgias (53,3%) (Tabla 2).

**Tabla 2:** Sintomatología y antecedentes de las madres con diagnóstico de zika (n=30).

Variable	n	%
Sintomatología		
Artralgia		
Sí	17	56,7
No	13	43,3
*Rash*		
Leve	10	33,3
Moderado	6	20,0
Severo	13	43,3
No	1	3,4
Conjuntivitis		
Sí	10	33,3
No	20	66,7
Mialgia		
Sí	16	53,3
No	14	46,7
Antecedentes		
Preeclampsia		
Sí	2	6,7
No	28	93,3
Eclampsia		
Sí	0	0,0
No	30	100
Síndrome de HELLP		
Sí	0	0,0
No	30	100
Sífilis		
Sí	0	0,0
No	30	100
Herpes		
Sí	1	3,3
No	29	96,7

En el desarrollo, las áreas que presentan puntaje medio más alto son la cognitiva con 44 (DE: 10), personal/social con 42 (DE: 11) y comunicación con 37 (DE: 8,7) (Figura 1).

**Figura 1:** Puntajes por área del desarrollo acorde con la escala de Battelle

Según, los puntajes anteriores, se evidencia que, en dos de las cinco áreas, los niños en riesgo fueron más frecuentes que los niños con normalidad y fortaleza. Las áreas con mayor porcentaje en riesgo fueron la adaptativa (80%), motora (53%) y comunicación (43%). Solo el 3% de los participantes presentó fortalezas en el área cognitiva (Material suplementario).

La evaluación del desarrollo mostró que 25 niños presentaban retraso en una de las cinco áreas evaluadas. La media de edad equivalente para las áreas motora, adaptativa, comunicativa, personal/social y cognitiva, respectivamente fue: 25,8 (DE: 7,8) meses, 26,7 (DE: 5,8) meses, 30,2 (DE: 6,9) meses, 33,5 (DE: 8,3) meses y 35,6 (DE: 5,9) meses. Se observó mayor compromiso en el dominio adaptativo y menor retraso en el dominio cognitivo (Tabla 3).

**Tabla 3:** Descripción y promedio de la edad en meses por cada área de acuerdo con los resultados de la escala Battelle (n=30).

Edad actual (meses)	Cognición	Comunicación	Motora	Adaptativa	Personal/social
	(edad equivalente en meses)	(edad equivalente en meses)	(edad equivalente en meses)	(edad equivalente en meses)	(edad equivalente en meses)
45	46	39	42	26	46
41	23	7	8	10	12
41	41	36	40	33	41
41	42	39	37	33	42
40	38	39	30	25	44
40	43	31	25	24	39
40	35	27	24	30	38
39	22	22	14	22	22
39	38	36	27	33	39
39	39	23	31	26	33
38	42	36	25	30	33
38	36	33	25	28	35
38	33	27	25	30	24
38	35	35	26	26	36
38	37	34	34	22	35
37	36	35	24	32	37
37	35	38	17	25	31
37	36	29	29	26	37
37	33	28	17	23	30
37	31	22	24	25	26
37	36	32	27	26	36
36	34	33	29	34	27
36	35	32	31	27	30
35	44	24	29	34	39
35	37	27	26	28	39
34	35	32	17	24	40
34	20	21	8	11	12
33	31	25	26	34	28
32	37	32	28	31	33
32	37	32	29	24	43
37,4 (2,9)^a^	35,6 (5,9)^a^	30,2 (6,9)^a^	25,8 (7,8)^a^	26,7 (5,8)^a^	33,5 (8,3)^a^

a Media y desviación estándar

## DISCUSIÓN

El estudio describe el estado del neurodesarrollo de 30 niños a los 36 meses de exposición al virus del Zika. Los puntajes sugieren que en promedio los niños presentaron una edad equivalente en meses inferior a su edad cronológica actual. Acorde con los puntajes, el 80% de los niños en el área adaptativa presentó el mayor riesgo de desarrollo.

Debilidades en el desarrollo motor y adaptativo significa que los niños, tres años después del nacimiento, tienen dificultades para ser independientes, asumir la responsabilidad de sus actos y en el control motor grueso y fino. Ambos aspectos son interdependientes, ya que la capacidad motora permite una mayor independencia y responsabilidad ^14^. Creemos, que está relacionado con la falta de habilidades y recursos de las madres para estimular a sus hijos, una vez conocido el diagnóstico. Lo anterior, debido a que la integración sensorio motriz, la estimulación motora gruesa y fina, depende del acompañamiento y retroalimentación que el cuidador puede ofrecer, junto a la intervención especializada de fisioterapia o terapeuta ocupacional que, en nuestro caso, es tardía ^7^^,^^9^. 

Sin embargo, el riesgo reportado en el área motora parece no afecta al rendimiento cognitivo de los participantes. Creemos que, el poco efecto que tiene el virus sobre la dimensión cognitiva (formación de categorías y conceptos), se debe a las actividades de estimulación accesibles en la interacción madre-hijo, como: la discriminación perceptiva, identificar y resolver problemas, valorar contradicciones e incoherencias ^15^^)^ que proponen la mayoría de los juegos para niños de 0 a 5 años ^16^.

La escala de Battelle ha sido adaptada culturalmente en Colombia, con alta confiabilidad interobservador ^13^. Ha sido utilizado en Colombia, donde tiene adecuadas propiedades psicométricas ^(^^17^. Si bien, es uno entre varias opciones de medición del neurodesarrollo, al ser una medida de alto interés clínico con múltiples aplicaciones prácticas, es susceptible de ser comparado con otros instrumentos que evalúan el mismo constructo, además cada una cuenta con puntos de corte amplios que permite dar una identificación adecuada del neurodesarrollo ^18^. Nuestros resultados comparados con otros estudios con otras patologías sugieren un grave impacto en el desarrollo asociado al zika ^13^^,^^14^.

Si bien, reconocemos que son necesarios estudios con diseños tipo casos control o cohortes, para profundizar sobre el efecto de la asociación entre las alteraciones en el neurodesarrollo y el zika, nuestros resultados descriptivos muestran alteraciones en los niños expuestos. En la evaluación del neurodesarrollo es importante considerar el tiempo de medición, nosotros evaluamos a los 36 meses, con resultados diferentes a estudios que consideraron 24 meses ^19^, en donde se encontró un comportamiento dispar en las dimensiones. Por ejemplo, se hallaron asociaciones significativas entre la exposición materna y el zika en las áreas personal/social y resolución de problemas, pero la comparación entre niños expuestos y no expuestos al virus, presentó niveles similares de desarrollo motor, resolución de problemas y habilidades personales y sociales. Incluso, el dominio del lenguaje fue más afectado en niños no expuestos al virus en comparación con los expuestos: 20,3% frente a los no expuestos 8,3% ^16^. Esta contradicción subraya la idea de la interacción entre dimensiones y el factor ambiental sobre la maduración cerebral ^20^.

Una limitación del estudio está relacionada con la falta de información desde el diagnóstico hasta la evolución del neurodesarrollo posterior, debido a que las características ambientales (nivel socioeconómico, ingresos, estilos de crianza, estimulación o deprivación afectiva o cultural, educación formal) interactúan con los factores biológicos y las características del individuo para producir las medidas de resultado evaluadas por el instrumento. Otra limitación fue la alta movilidad social de la población colombiana, ya que, de los 122 niños registrados en la base de datos, solo fue posible contactar a 30. Sin embargo, nuestra fortaleza es que este es uno de los pocos estudios que realiza la valoración del neurodesarrollo en pacientes expuestos al virus del Zika en Colombia. Sin embargo, reconocemos que la presencia de un grupo de control hubiese permitido evaluar el neurodesarrollo en condiciones sociodemográficas, nutricionales, culturales y familiares similares al grupo evaluado, lo que favorecería observar el papel del virus sobre el neurodesarrollo. No obstante, encontramos estudios que exploran el neurodesarrollo asociado al virus, sin grupo de control y con un bajo número de casos ^21^^,^^22^.

De igual forma, el neurodesarrollo es un fenómeno multicausal y complejo en el que interactúan aspectos biológicos y socioculturales. Desconocemos en nuestra muestra algunas características biológicas como el estado nutricional, parasitosis intestinal y malaria que afectan el neurodesarrollo. A su vez, aspectos socioculturales como la estimulación educativa o parental no fueron controlados en el estudio y estas características contribuyen a explicar el estado de desarrollo del grupo de niños evaluados, por tanto, el neurodesarrollo está afectado por las características descritas; por esta razón, nuestros resultados deben interpretarse con estas limitaciones. Sin embargo, estudios comparables de seguimiento de niños expuestos al virus del Zika toman variables similares (variables antropométricas, peso actual, peso al nacer, talla actual y talla al nacer), consideradas como una medida de las condiciones biológicas del niño al momento de la evaluación ^22^^,^^23^.

Un factor que puede modular el neurodesarrollo motor es el incremento del uso de dispositivos digitales en nuestra época. Por ejemplo, a menor edad de uso de dispositivos digitales, mayores errores en tareas de desempeño motor, sin embargo, esta relación es poco significativa debido al efecto de la edad que modula la relación entre el uso del dispositivo digital y el desarrollo neuromotor ^24^, por ello, sería recomendable hacer un seguimiento longitudinal de los niños tomando en cuenta el uso del dispositivo móvil, características de la estimulación, estado nutricional y condiciones clínicas reportadas y la edad.

En nuestro estudio, los niños expuestos presentaron un desarrollo insuficiente al tercer año de vida. Dado que este estudio describe el desarrollo de niños expuestos en Colombia, se hace necesario realizar estudios de seguimiento que permitan corroborar estos hallazgos. El diagnóstico temprano en la gestante permitirá intervenciones tempranas del sistema de salud, focalizado en poblaciones donde hay brotes por el virus. Ello, depende de la oportunidad del diagnóstico, debido al corto período para la detección de virus, siendo necesario contar con pruebas serológicas con resultados tempranos. El seguimiento durante la primera infancia es crucial para detectar posibles anomalías neurológicas, las cuales se pueden manifestar en las consultas de atención primaria, las intervenciones tempranas por especialistas pueden evitar repercusiones en el neurodesarrollo.

Aunque el número de casos de zika ha disminuido significativamente, la infección continúa propagándose en algunos países de las Américas ^21^. Como resultado, es probable que los servicios de salud se sobrecarguen, en áreas donde todavía existe un alto número de casos. Es importante que los cuidadores tengan acceso a herramientas de detección del desarrollo neurológico en zonas donde los sistemas de salud tienen menos acceso.

En conclusión, el neurodesarrollo de 30 niños expuestos intrauterinamente al virus del Zika, mostró un retraso en el neurodesarrollo, en particular en las áreas motora y adaptativa. Si bien el neurodesarrollo es un proceso de integración e interacción entre dimensiones biológicas y ambientales, los niños expuestos al zika en condiciones socioeconómicas bajas, se recomienda orientar las intervenciones por parte de cuidadores y personal de salud en el fortalecimiento de destrezas motoras y adaptativas, las cuales son dimensiones del neurodesarrollo que posibilitan la exploración y autonomía del niño.
